# In Vitro and In Vivo Miltefosine Susceptibility of a *Leishmania amazonensis* Isolate from a Patient with Diffuse Cutaneous Leishmaniasis: Follow-Up

**DOI:** 10.1371/journal.pntd.0004720

**Published:** 2016-07-14

**Authors:** Adriano C. Coelho, Cristiana T. Trinconi, Carlos H. N. Costa, Silvia R. B. Uliana

**Affiliations:** 1 Departamento de Parasitologia, Instituto de Ciências Biomédicas, Universidade de São Paulo, São Paulo, São Paulo, Brazil; 2 Departamento de Medicina Comunitária, Universidade Federal do Piauí, Teresina, Piauí, Brazil; Hebrew University-Hadassah Medical School, ISRAEL

Miltefosine is the most efficacious oral drug available for the treatment of leishmaniasis. While miltefosine has been in use in India for the treatment of visceral leishmaniasis for a number of years [1], its application to the treatment of cutaneous leishmaniasis is still under investigation [2–4]. We recently reported that miltefosine was effective in the treatment of *Leishmania amazonensis* infections in a mouse experimental model [5]. Those results were obtained in BALB/c mice infected with two different *L*. *amazonensis* strains: a type strain (M2269) and an isolate obtained from a patient with diffuse cutaneous leishmaniasis (2506). Five weeks after infection, treatment was initiated when lesions on the hind left footpad were apparent. Miltefosine 13 mg/kg/day was given orally for 15 consecutive days to groups composed of five mice. The animals infected with M2269 or 2506 parasites were considered clinically cured at the end of treatment. One week after the end of treatment, the mice were humanely killed for parasitological examination. Histopathological examinations of the infection site and limiting dilution assays performed with the footpad tissue revealed an absence of parasites [5]. Clinical follow-up for 15 weeks after the end of the treatment showed no signs of relapse. These results were considered as evidence that treatment with miltefosine in this murine model of cutaneous leishmaniasis, a model of extreme susceptibility, was highly efficacious.

Subsequently, one of the groups described, consisting of four remaining mice infected with the M2269 strain and treated with the same dose of miltefosine, was followed up for a longer period. These animals relapsed from the 23rd week post-infection. Lesions became apparent in all four mice and appeared individually in weeks 23, 36, 39, and 39 post-infection. At these times, parasites were recovered from lesions, and the susceptibility to miltefosine was determined, as described by Coelho et al. [5]. No significant changes in drug susceptibility were found, indicating that there was no acquired resistance to miltefosine during therapy.

Furthermore, we reported that the diffuse cutaneous leishmaniasis patient from which the 2506 strain had been isolated was responding well to miltefosine and pentamidine combined therapy [5]. However, after 18 months the disease reoccurred, requiring a new course of treatment. Previous clinical studies also described the occurrence of relapses after miltefosine chemotherapy in patients with diffuse cutaneous leishmaniasis [6,7].

Parasite persistence after treatment with various drugs has been described [8–11] and may, in fact, be the rule in leishmaniasis. The relapses observed in this group of mice indicate that the techniques used in our previous report to characterize the parasite burden (histopathology and limiting dilution) lack the sensitivity to detect these remaining or quiescent parasites. We believe these findings are significant to the data previously reported.
